# Switching between global and local levels: the level repetition effect and its hemispheric asymmetry

**DOI:** 10.3389/fpsyg.2014.00252

**Published:** 2014-03-25

**Authors:** Luc Kéïta, Nathalie Bedoin, Jacob A. Burack, Franco Lepore

**Affiliations:** ^1^Département de Psychologie, Université de MontréalMontréal, QC, Canada; ^2^Laboratoire Dynamique du Langage, UMR CNRS 5596, Université Lyon 2France; ^3^Department of Educational and Counselling Psychology, McGill University, MontréalQC, Canada

**Keywords:** hemispheric asymetry, hierarchical stimuli, switching, level repetition, inhibition

## Abstract

The global level of hierarchical stimuli (Navon’s stimuli) is typically processed quicker and better than the local level; further differential hemispheric dominance is described for local (left hemisphere, LH) and global (right hemisphere, RH) processing. However, neuroimaging and behavioral data indicate that stimulus category (letter or object) could modulate the hemispheric asymmetry for the local level processing. Besides, when the targets are unpredictably displayed at the global or local level, the participant has to switch between levels, and the magnitude of the switch cost increases with the number of repeated-level trials preceding the switch. The hemispheric asymmetries associated with level switching is an unresolved issue. LH areas may be involved in carrying over the target level information in case of level repetition. These areas may also largely participate in the processing of level-changed trials. Here we hypothesized that RH areas underly the inhibitory mechanism performed on the irrelevant level, as one of the components of the level switching process. In an experiment using a within-subject design, hierarchical stimuli were briefly presented either to the right or to the left visual field. 32 adults were instructed to identify the target at the global or local level. We assessed a possible RH dominance for the non-target level inhibition by varying the attentional demands through the manipulation of level repetitions (two or gour repeated-level trials before the switch). The behavioral data confirmed a LH specialization only for the local level processing of letter-based stimuli, and detrimental effect of increased level repetitions before a switch. Further, data provides evidence for a RH advantage in inhibiting the non-target level. Taken together, the data supports the notion of the existence of multiple mechanisms underlying level-switch effects.

## INTRODUCTION

Visual processing of global and local features of objects has been widely investigated with hierarchically organized stimuli (Navon, 1977), which are large (global) letters made up of mutually identical small (local) letters. These stimuli are thought to provide an experimental simplification of the complex multilevel natural visual environment (List et al., 2013). A functional hemispheric asymmetry is classically reflected by a right hemisphere (RH) advantage for global processing and a left hemisphere (LH) advantage for local processing. This notion is supported by extensive evidence from brain damaged patients (Robertson et al., 1988; Lamb et al., 1990; Robertson and Lamb, 1991), brain imagery investigations (Fink et al., 1996; Martinez et al., 1997; Han et al., 2000), event-related potentials (ERP) studies (Grabowska and Nowicka, 1996; Proverbio et al., 1998; Evans et al., 2000) and behavioral experiments involving lateralised presentation of compound stimuli (Blanca et al., 1994; Hübner, 1997; Evert and Kmen, 2003; Hübner et al., 2007). according to ERP data, the hierarchical processing modulates activities in the visual cortex at latencies as short as 110 ms (Han et al., 2000). In the early visual (prestriate) processing areas, attention to the global or local levels is respectively associated with activations in the right lingual gyrus and the left inferior occipital cortex (Fink et al., 1996). This asymmetry is also observed in higher level processing areas, which may mediate the voluntary distribution of selective attention across the complexity levels (Rafal and Robertson, 1995) and modulate computations performed in the prestriate cortex (Fink et al., 1996). This is consistent with evidence of impaired global processing in patients with right temporal-parietal lesions, but impaired local processing with left temporal-parietal lesions (Robertson et al., 1988; Robertson and Lamb, 1991).

An alternative approach is that the hemispheric asymmetry for local level processing is modulated by the stimulus category as the classical hemispheric asymmetry for global/local processing is not observed when the hierarchical stimuli are not made of alphabetic material, (Bedson and Turnbull, 2002). According to both positron emission tomography (PET) data (Fink et al., 1996, 1997b) and to behavioral findings from experiments with visual half-field presentation (Keita and Bedoin, 2011), RH dominance can be observed for the local processing of object-based hierarchical stimuli when the stimulus category (letter vs. object) is known in advance. According to the lateralisation of cerebral networks specialized for the stimulus category, the highly demanding local level processing is assumed to engage one hemisphere more than the other.

In contrast to the demands of selective attention paradigms in which attention is focused at one level of complexity, targets in divided attention paradigms are equiprobably but unpredictably displayed either at the global or local level. Decreased performance is then observed for changed-level as compared to repeated-level trials (Ward, 1982; Robertson, 1996; Lamb et al., 1999), an effect which has been dissociated from response- and stimulus-changing effects (Robertson, 1996; Filoteo et al., 2001; List et al., 2013). This difference may be due to attentional processes, as the advantage for repeated-level trials may reflect a level-specific priming effect and the carry-over of target level information from the last trial may involve the left inferior parietal lobe. Conversely, decreased performance for changed-level trials may relate to additional attention switching performed between the two processing modes. The switch-cost is independent of the resolution or the actual size of the targets (Fink et al., 1996; Kim et al., 1999; Filoteo et al., 2001) and is not strictly based on a change in the selection of spatial frequencies (Lamb and Yund, 1996a). Therefore, switching between levels within a hierarchical stimulus is not strictly based on a zoom lens of attention, but also on changes in the attentional weights associated with each level (Robertson, 1996).

When considered as a unitary mechanism, switching between levels is often described as an executive attentional mechanism mainly based on LH areas. Its neural bases have been assessed by increasing the overall demands imposed on this process. These demands are increased when a switch trial is separated from the last switch by a small length of time (Wilkinson et al., 2001), and when a high number of level-changed trials occur in an experiment (Fink et al., 1997a), which is associated with activations in the precuneus, the left supplementary motor area, and the left medial parietal areas. This is consistent with ERP evidence that a positive potential peaking at 290 ms over the left parietal and left posterior temporal regions was higher for changed-level trials than for repeated-level trials (Schatz and Erlandson, 2003). However, the neuropsychological evidence is mixed. An impairment of global/local level-switches have been described in cases of both left dorsal parietal lesions (Rafal and Robertson, 1995) and right temporal-parietal lobe damage (Filoteo et al., 2001). This suggests the involvement of this right cortical area in monitoring attentional weights to different hierarchical levels for switch trials.

Varying the number of repeated-level trials before a changed-level trial may specifically modulate the demands for the inhibition of the inappropriate level of analysis. For example, the magnitude of the switch cost has been shown to increase with more targets identified at the same level before the switch (Wilkinson et al., 2001). Compared with a level switch performed after two repeated-level trials, a switch performed after four or six repeated-level trials is associated with bilateral activation of a parietal-motor area, which suggests that right lateralised are crucial for inhibiting the inappropriate level of analysis.

The current study was aimed to replicate the modulation of hemispheric asymmetry for the local level processing of hierarchical stimuli by the stimulus category. Therefore, better performance was expected for local targets presented in the right visual field (RVF-LH) than in the left visual field (LVF-RH) only for letter-based hierarchical stimuli. As this effect has been observed only in between-subject comparisons, we sought to replicate it with a within-subject design in a divided attention task. The experiment was also designed to test the prominent involvement of right cerebral areas in inhibiting the inappropriate processing level when performing an intra-stimulus (hierarchical) switch between levels. Changed-level trials were presented after either two or four repeated-level trials to modulate the demands on this inhibitory process. We expected switching after four repeated-level trials to require inhibitory processing and therefore to be better performed in the LVF-RH than in the RVF-LH. We assess the detrimental effect of response changing by comparing a no-change condition (the visual field, the target level, and the response were the same as in the preceding trial) with a changed-response condition (the only difference with the previous trial was the target (i.e., the response). In contrast to the cost of switching, the cost of response changing was not expected to be lower for the targets displayed to the LVF-RH, which therebyhighlights the specificity of inhibiting the irrelevant complexity level as one of the mechanisms underlying the between-level switching process.

## MATERIALS AND METHODS

### PARTICIPANTS

Thirty-two university students (22 female and 12 male; mean age = 22.8 years, +3.3) performed both the letter block and the object block tasks. All the participants had normal or corrected-to-normal vision and were strongly right-handed (9 or 10 right-handed responses out of a total of 10 of the most reliable items of the Edinburgh Handedness Inventory). They gave informed and written consent to participate.

### STIMULI

**FIGURE 1:**
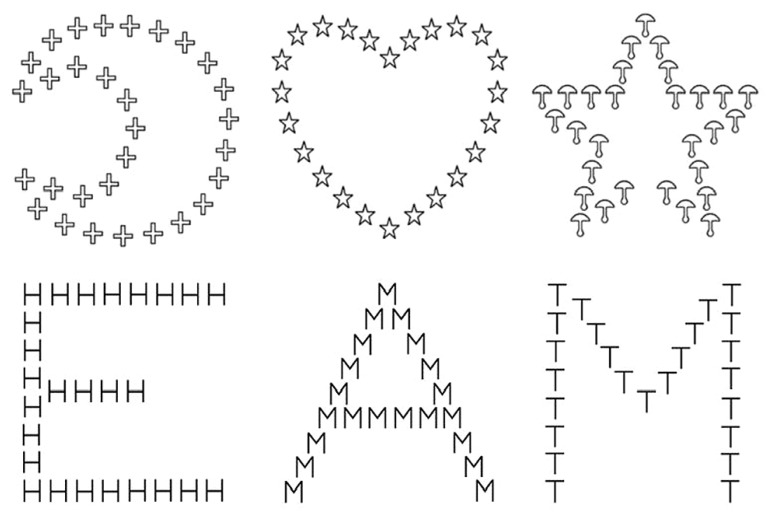
**Examples of figures used as object-based hierarchical stimuli with the star and the moon as the targets, xand letter-based hierarchical stimuli with E and M as the target letters**.

The stimuli included a block of hierarchical letters (letter block) and a block of hierarchical objects (object block) drawn in black on a white background. Their order of presentation was counterbalanced across participants. The hierarchical letters were each a large (global) letter made up of smaller (local) letters (**Figure 1**). Global and local letters always differed within a hierarchical stimulus. One of the two targets (E or M) was located either at the local or the global level, while the distractor letters (H, T, or A) were presented at the other level. The 96 experimental trials were equally displayed either to the RVF-LH or to the LVF-RH. In each hemifield, the target appeared at the local level in half of the trials and at the global level in the other half. The presentations of E and M were equally likely both in each of the four level by field combinations and in being associated with each of the three distractor letters. In the letter block, we used 120 filler trials, which each involved one of the target letters. The side of presentation and the target level of the filler trials were equated following the same rules as for the experimental trials. The global letter subtended 3.8°(horizontal) × 4.0°(vertical) of visual angle; the local letter subtended 0.35° (horizontal) × 0.4° (vertical) of visual angle and were separated by 0.1°.

The object block also included 96 experimental trials and 120 filler trials. The objects presented at the global and the local levels always differed within a hierarchical stimulus. One of the two targets (star or moon) was displayed either at the local or global level, while a distractor object (mushroom, cross, or heart) was presented at the other level (**Figure 1**). The drawings were as simple as possible, but the shape of the objects was slightly more complex than those of the letters as they were made up of 24 to 32 elements, while the letters were made up of 16 to 26 elements. The size of the local and global objects was the same as the size of the local and global letters, with the same spacing between the elements. The same rules as in the letter block were applied regarding the presentation of the 96 experimental trials and the 120 filler trials for the level, the visual field, and the combination of targets with the distractor objects.

### GENERAL PROCEDURE

Each participant was tested individually in a sound attenuated booth and sat in front of an Apple Macintosh iBook at a constant distance of 57 cm from the screen. At the beginning of each trial, a fixation point (=) appeared at the center of the screen for 1500 ms. The hierarchical stimulus was displayed during the last 175 ms of the display of the fixation point, either to the RVF or the LVF. Its nearest border was 2° distant from the fixation point. The filler trials were distributed through the list to avoid any regularity within the presentation sequence. The sequence consisted of 1–3 consecutive left or right displays, 1–5 consecutive identical targets, and 1–4 repeated-level trials to prevent participants from learning any rules regarding the following stimulus.

**FIGURE 2:**
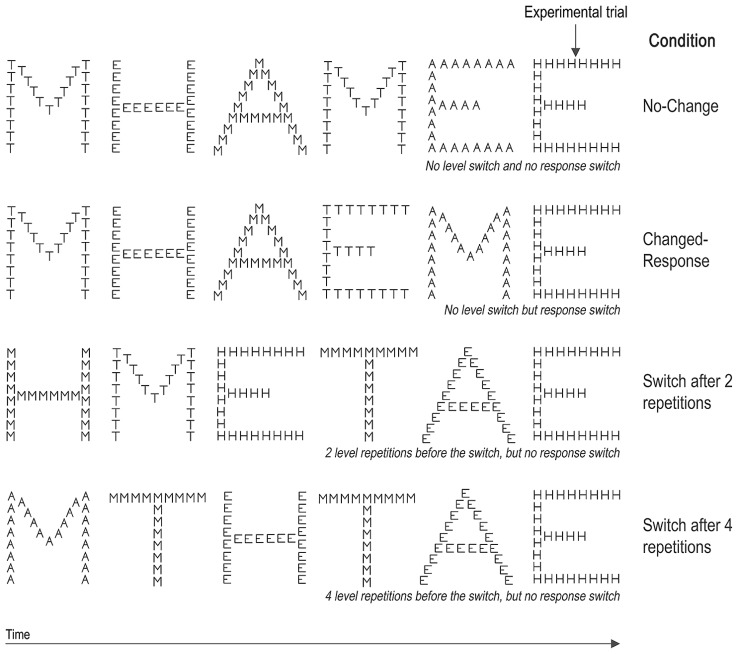
**Examples of context stimuli sequences regarding each of the context conditions**.

The 192 letter- and object-based experimental trials were equally distributed among four conditions. The changed-level trials appeared either after two (48 trials) or four (48 trials) repeated-level trials. The stimuli presented in the no-change condition (48 trials) were preceded by two repeated-level trials. To avoid confounding the level-switch cost and the costs due to response changing or to spatial shifting, the experimental trials in both changed-level conditions and in the no-change condition always followed a trial displayed to the same hemifield and containing the same target. In the no-change condition, the target in *n – *1 and *n – *2 were located at the same level as the *n* target. To assess the cost associated with reponse changing, performance in the no-change condition was compared with performance in the changed-response condition (48 trials). In the changed-response condition, the *n* trial was preceded by two repeated-level trials and *n - 1* was displayed in the same hemifield but it contained a different target (see **Figure 2** for examples of the four context conditions).

The task was to decide whether the hierarchical stimulus contained E or M (in the letter block) and the moon or the star (in the object block). The participants were asked to respond as quickly and accurately as possible by pressing one of the two associated keys with the left or right index finger. In each hemifield, the same proportion of trials required left and right index responses, in order to maintain the same probability of a stimulus-response compatibility (Simon effect) to occur in each experimental condition. The next trial began 1500 ms after the response. Response times (RT) and accuracy were recorded for each trial. A rest period was proposed between the two blocks, and each of them began with 12 practice trials. Each block was punctuated with a break.

### DATA ANALYSIS

Mean RTs for correct responses and errors rate (ERs) were analyzed using four-factor repeated-measure ANOVAs with a Greenhouse-Geisser correction, with four within-subject factors: category (letter, object), level (global, local), visual field (RVF-LH, LVF-LH), and context (no-change, changed-response, switch after two level repetitions, switch after 4 level repetitions). Contrasts were reported regarding the expected differences between conditions. The alpha level was set at 0.05. The effect size was estimated by calculating partial eta-squares ($\eta_{p}^{2}$) and, in accordance with Cohen (1988), it was considered as small if $\eta_{p}^{2}$ = 0.01, medium if $\eta_{p}^{2}$ = 0.06, and large if $\eta_{p}^{2}$ = 0.14.

## RESULTS

**FIGURE 3:**
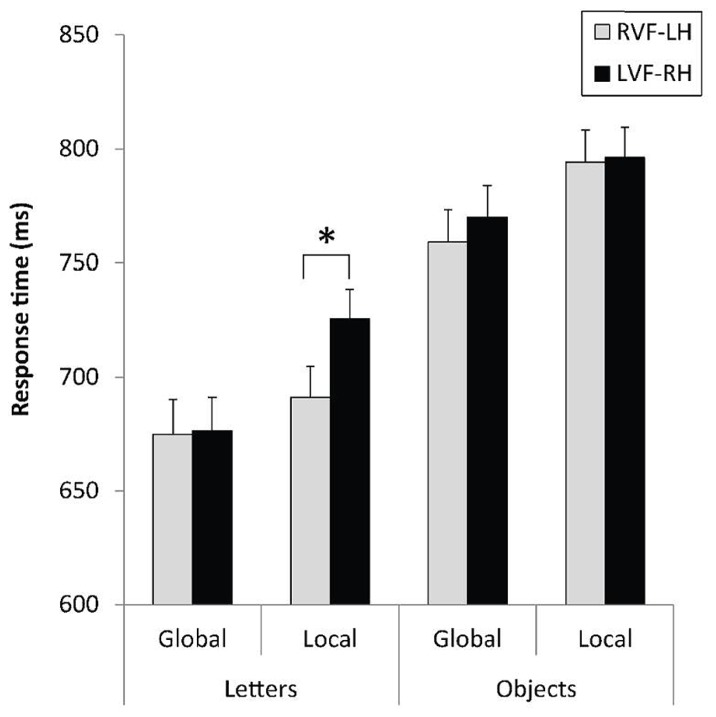
**Mean response times and standard errors for letter-based and object-based hierarchical stimuli displayed to the right (RVF-LH) or left visual field (LVF-RH), as a function of the level of the target (global, local).** *p < 0.05.

The analysis revealed a main effect of category with shorter RTs, *F*(1,31) = 20.96, *p* < 0.0001, $\eta_{p}^{2}$ = 0.40, and better response accuracy, * F*(1,31) = 23.53, *p* < 0.0001, $\eta_{p}^{2}$ = 0.43, for letter-based than for object-based stimuli. A main effect of level was also observed, as indexed by shorter latencies, *F*(1,31) = 13.16, *p* < 0.001, $\eta_{p}^{2}$ = 0.30, and fewer errors, *F*(1,31) = 7.72, *p* < 0.01, $\eta_{p}^{2}$ = 0.20, for the global than for the local level. The level × field × category interaction was obtained on RTs, *F*(1,31) = 4.43, *p* < 0.044, $\eta_{p}^{2}$ = 0.013, and constrasts indicated the expected difference in hemispheric asymmetry for the local level according to the stimulus category. As illustrated in **Figure 3**, the identification of local target letters was faster in the RVF-LH than in the LVF-RH, *F*(1,31) = 11.86, *p* < 0.002, $\eta_{p}^{2}$ = 0.03, whereas this index of LH dominance for local processing disappeared for object-based stimuli, *F*(1,31) < 1.

The visual field effects (VFE = LVF – RVF) are presented in **Table 1**. As for local targets, the VFE was significantly lower for object-based than for letter-based stimuli, *F*(1,31) = 5.29, *p* = 0.0283, $\eta_{p}^{2}$ = 0.15.

**Table 1:** Mean response times in milliseconds and errors rates (standard errors in parenthesis) across visual field conditions.

		Letter-based stimuli		Object-based stimuli	
Target level		Global	Local	Global	Local
RVF-LH	TR	675 (15.49)	691 (13.96)	759 (14.34)	794 (14.16)
	ER (%)	2.47 (0.53)	3.52 (0.68)	6.77 (0.93)	6.77 (1.04)
LVF-RH	TR	676 (14.82)	725 (12.99)	770 (14.10)	796 (13.47)
	ER (%)	1.17 (0.42)	5.73 (0.88)	5.34 (0.87)	6.25 (0.96)
Difference: LVF-RVF	TR	1	34	11	2
	ER (%)	-1.3	2.21	-1.43	-0.52

*RVF-LH, right visual field-left hemisphere; LVF-RH, left visual field-right hemisphere.*

**FIGURE 4:**
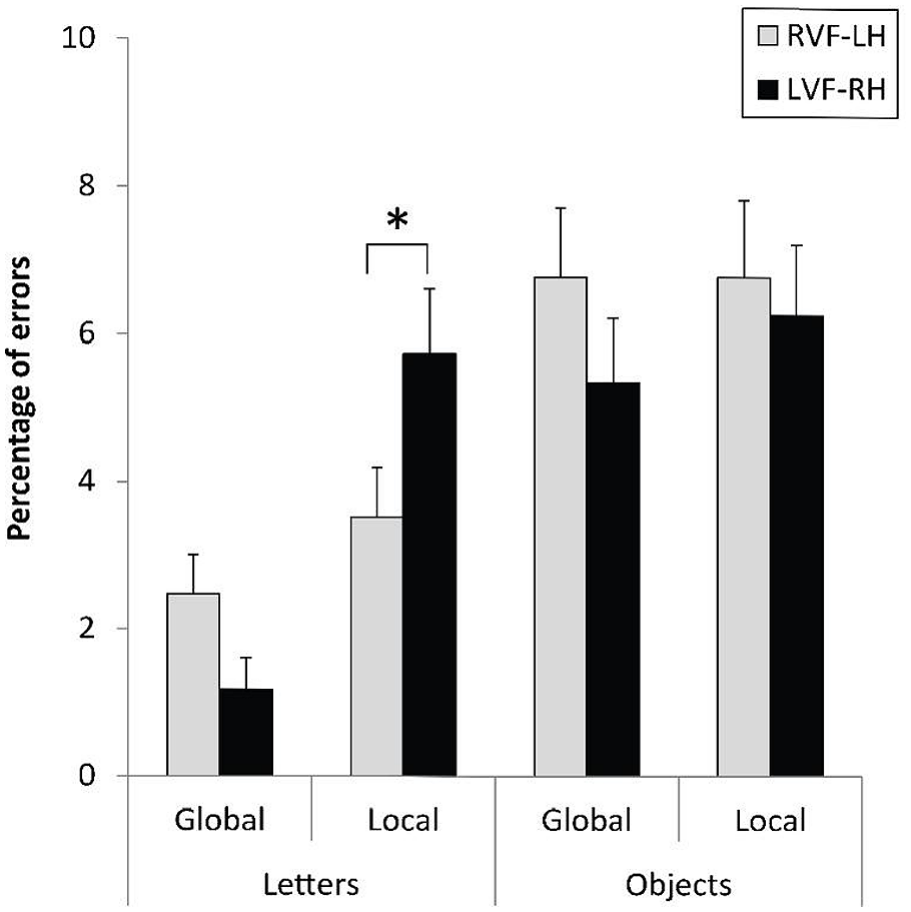
**Mean error rates and standard errors for letter-based and object-based hierarchical stimuli displayed to the right (RVF-LH) or left visual field (LVF-RH), as a function of the level of the target (global, local).** *p < 0.05.

The level × field × category interaction did not reach significance with ERs, *F*(1,31) = 2.16, *p* = 0.15. However, any phenomenon of speed-accuracy trade-off can be excluded according to the pattern of results observed in **Figure 4**. A RVF-LH advantage was indeed recorded for the local letters, *F*(1,31) = 6.24, *p* < 0.019, $\eta_{p}^{2}$ = 0.06, while no hemispheric asymmetry occurred for response accuracy regarding the local processing of hierarchical objects, *F*(1,31) < 1. The VFE was significantly higher on error rates for local letters than for local objects, *F*(1,31) = 4.76, *p* = 0.0368, $\eta_{p}^{2}$ = 0.13.

**FIGURE 5:**
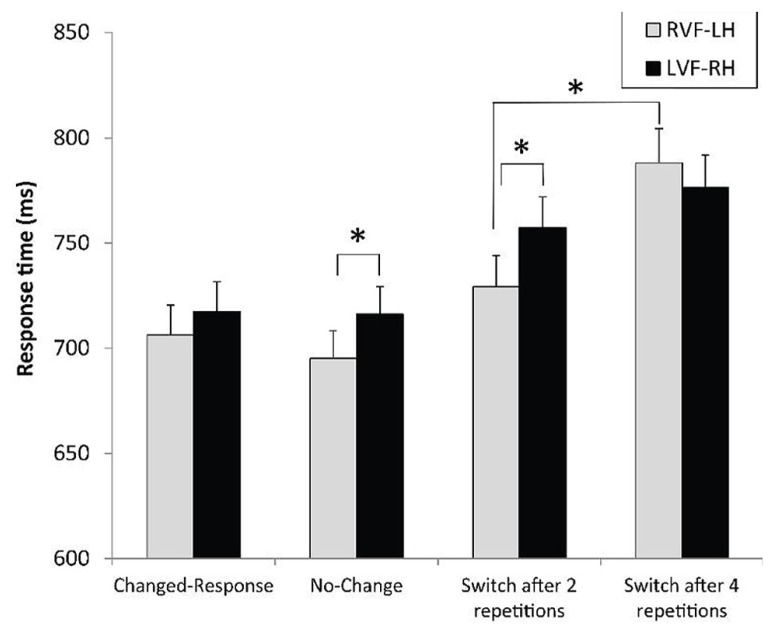
**Mean response times and standard errors for letter-based and object-based hierarchical stimuli according to the context preceding the experimental trial and the visual field of presentation.** *p < 0.05.

**FIGURE 6:**
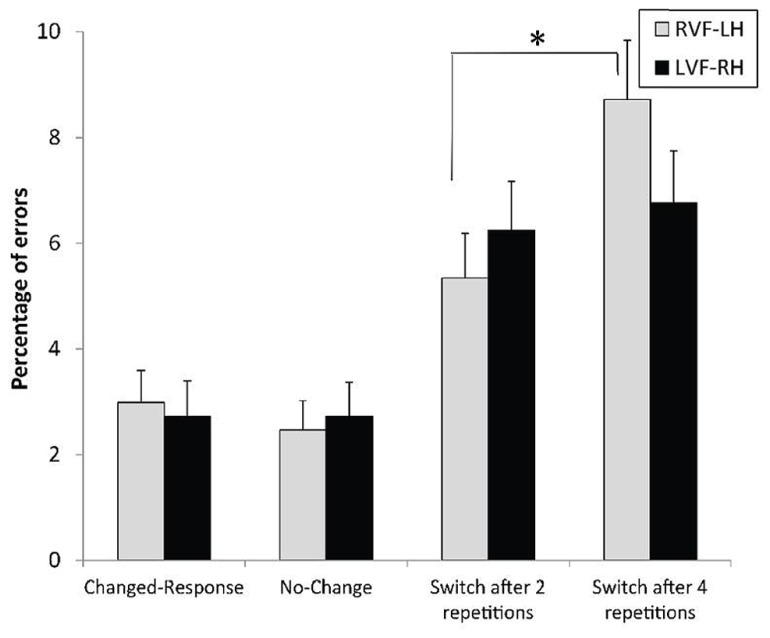
**Mean error rates and standard errors, according to the context preceding the experimental trial and the visual field of presentation.** *p < 0.05.

We obtained a main effect of context with RTs, *F*(3,93) = 33.57, *p* < 0.0001, $\eta_{p}^{2}$ = 0.52, which could not be explained by the cost due to switching between responses, since the comparison between the no-change and the changed-response conditions was not significant, *F*(1,93) < 1 (**Figure 5**). However, as predicted, the main effect of context reflected the dramatic increase in response latency with the necessity to switch between levels, as confirmed by the difference between no-change and switch after two repetitions conditions, *F*(1,93) = 19.40, *p* < 0.0001, $\eta_{p}^{2}$= 0.17. Additionally, RTs for changed-level trials were significantly longer after four rather than two repeated-level trials, * F*(1,93) = 20.63, *p *< 0.0001, $\eta_{p}^{2}$ = 0.18. The analysis of ERs confirmed the main context effect, *F*(3,93) = 13.87, *p* < 0.0001, $\eta_{p}^{2}$ = 0.31, and the lack of significant cost due to switching between responses, *F*(1,93) < 1 (**Figure 6**). Consistent with the pattern of results on RTs, the level-switch cost was observed with ERs, *F*(1,93) = 11.61, * p* < 0.001, $\eta_{p}^{2}$ = 0.11, as was the detrimental effect of increased number of level repetitions before a level switch, *F*(1,93) = 4.35, *p* < 0.04, $\eta_{p}^{2}$ = 0.04.

Regarding hemispheric asymmetry, a context × field interaction was observed with RTs, *F*(3,93) = 2.91, *p* < 0.039, $\eta_{p}^{2}$ = 0.09, indicating two phenomena (**Figure 5**). One, the RVF-LH advantage was much higher when a switch occurred after two level repetitions, *F*(1,93) = 7.60, *p* < 0.007, $\eta_{p}^{2}$ = 0.08, than in the no-change condition, *F*(1,93) = 4.23, *p* < 0.043, $\eta_{p}^{2}$ = 0.04. Two, the detrimental effect of the increased number of level repetitions before switching was significant for targets displayed to the RVF-LH, *F*(1,93) = 32.69, *p* < 0.0001, $\eta_{p}^{2}$ = 0.26, but not for targets displayed to the LVF-RH, *F*(1,93) = 3.25, *p *= 0.08. Similarly, the VFE significantly differed between switching after two or after four level repetitions, *F*(1,93) = 7.66, *p* = 0.0068, $\eta_{p}^{2}$ = 0.08 (**Table 2**).

**Table 2:** Mean response times in milliseconds and errors rates (standard errors in parenthesis) across context conditions.

Target level		Response-change	No-change	Switch after 2 repetitions	Switch after 4 repetitions
RVF-LH	TR	706 (14.37)	695 (13.44)	729 (14.90)	788 (16.32)
	% of ER	3.00 (0.60)	2.47 (0.56)	5.34 (0.85)	8.73 (1.11)
LVF-RH	TR	718 (13.89)	716 (12.77)	758 (14.59)	776 (15.65)
	% of ER	2.74 (0.66)	2.74 (0.63)	6.25 (0.92)	6.77 (0.97)
Difference: LVF-RVF	TR	12	21	29	-12
	ER (%)	-0.26	0.27	0.91	-1.96

*RVF-LH, right visual field-left hemisphere; LVF-RH, left visual field-right hemisphere.*

The analysis with ERs confirmed this pattern of results, but the context × field interaction did not reach significance, *F*(3,93) = 1.40, *p* = 0.24 (**Figure 6**). However, response accuracy in the changed-level conditions was significantly affected by the visual field in the expected direction.

The contrasts provided convergent evidence for a RH advantage in inhibiting the non-target level. One, switches required in the most difficult condition regarding the inhibitory process (switch after four repetitions) strongly tended to be more accurately performed for targets displayed to the LVF-RH than to the RVF-LH, *F*(1,93) = 3.56, *p* = 0.06, $\eta_{p}^{2}$ = 0.04. Similarly, the VFE stringly tended to differ after four and after two repeated levels, *F*(1,93) = 3.83, *p* = 0.0608, $\eta_{p}^{2}$ = 0.04. Two, parallel to the RT data, switches were negatively affected by the increased number of previous repeated-level repetitions in the RVF-LH, *F*(1,93) = 10.69, *p* < 0.002, $\eta_{p}^{2}$ = 0.10, but not in the LVF-RH, *F*(1,93) < 1.

## DISCUSSION

The goals of the study were to assess the modulation of hemispheric asymmetry for local processing of visual hierarchical information by the stimulus category and to selectively address inhibition of the inappropriate level of analysis as one of the specific components of the ability to switch between levels. We aimed to stress the implication of the RH in the inhibition process in switching after more repetitions of the target at the same level. Taken together, the results replicated the typical LH advantage for the identification of local targets in letter-based hierarchical stimuli. The second main finding of our study was that the increased number of level repetitions before a level switch was detrimental to the speed and the accuracy of the hierarchical stimulus processing in the RVF-LH, but not in the LVF-RH, suggesting that right-sided cerebral areas are much efficient in the inhibition mechanism involved in switching between levels.

### THE INFLUENCE OF STIMULUS CATEGORY ON HEMISPHERIC ASYMMETRY IN LOCAL PROCESSING

Many findings support the notion that the RH is more efficient in the global processing of compound stimuli, while the LH is biased toward attending to and processing its local elements. However, this has not been found in a number of studies with rapid lateralised presentations (Van Kleeck, 1989; Yovel et al., 2001). The results obtained in our experiment confirmed that the local processing of object-based hierarchical stimuli is not associated with the typical LH advantage. Therefore, the functional hemispheric asymmetry in perceptual processes may be modulated by higher order attentional “top-down” mechanisms due to characteristics of the task. These mechanisms probably rely on temporal-parietal areas and play a supervisory role in the attentional control for global/local processing within the prestriate cortex (Yamaguchi et al., 2000). For example, the classical hemispheric asymmetries for global/local processing are more robust in divided- than focused-attention tasks (Van Kleeck, 1989; Heinze et al., 1998; Yovel et al., 2001), and when solving information conflict between levels is necessary (Hübner and Malinowski, 2002; Malinowski et al., 2002; Volberg and Hubner, 2004, 2006; Hübner and Volberg, 2005; Hübner et al., 2007). Additionally, hemispheric asymmetries due to the global-local distinction can be obscured by some aspects of the material which may produce co-varying effects due to the involvement of other processes which are also lateralised.

The present findings support the notion that the category of information is one of the co-varying factors associated with hemispheric asymmetries in processing hierarchical stimuli, since LH dominance was obtained for local letters but not for local objects. We attemptedto modulate hemispheric asymmetry specially regarding the local level, which may impose greater perceptual demand on target identification (Fink et al., 1997b). To compensate for this difficulty, the local level of compound stimuli may engage additional mechanisms to improve the processing of small elements. Therefore, lateralised cognitive mechanisms may be engaged in the local processing either because they underly the processing of details or because they are specialized in the category of the stimulus content. This may result in the selective engagement of left- or right-sided areas in local target identification for hierarchical letters and hierarchical objects, respectively. This notion is supported by Bedson and Turnbull (2002) who also reported LH dominance in the case of local processing when the targets were letters only but not when they were shapeswhich had fewer less “linguistic” properties. The data here are consistent with this pattern of findings for both rapidity and accuracy of responses by using compound letters and compound object drawings.

Consistent with evidence of higher involvement of the RH areas for local processing of object-based hierarchical stimuli found with PET data (Fink et al., 1996, 1997a), we have previously found dominance of RH areas for local objects and LH for local letters with the same material and task as used in the current study but with a between-subject design (Keita and Bedoin, 2011). In the present experiment, the LH dominance for local processing disappeared in case of object-based hierarchical stimuli, but no RH dominance was actually observed. This lack of clues for RH dominance may be partly due to the within-subject design. Indeed, in a between-subject design, the participants respond to only one category of information (alphabetic vs. non-alphabetic) which may lead to assigning a value to the stimulus content, resulting in important modulation of hemispheric asymmetry by the category. In contrast, in the within-subject design used in the present experiment, the participants performed the task on letter-based and object-based hierarchical stimuli, which may reduce the importance devoted to the stimulus category.

The present findings also differed from those in our previous study in which a significant advantage for global targets was not observed, despite the global/local size ratio was the same in both studies. This global/local size ratio was chosen to get the same perceptual salience for local and global targets (Keita and Bedoin, 2011). The reason for the advantage for global targets in the current experiment is unclear, but the evidence suggests that attention was biased toward this level. The ability to select information against dominant information (here, the ability to select the local level) has been shown to rely on the left inferior parietal cortex (Mevorach et al., 2006), and the involvement of this LH area could contribute to mask the effect of the RH involvement in the local processing of object-based compound stimuli.

### THE RIGHT HEMISPHERE INVOLVEMENT IN INHIBITION DURING SWITCHING

The difference in performance between the no-change trials and the changed-trials after two repetitions replicated the detrimental effect of switching on performance (Robertson, 1996). Previous evidence of its dissociations from response- and stimulus-changing effects (Robertson, 1996; Filoteo et al., 2001; List et al., 2013) is consistent with the findings here that switching between levels more dramatically decreased performance than changing motor responses between successive trials. Thus, this process appears to impose considerable demands on cognitive resources. The findings also indicate that a switch between levels which presents moderate difficulty (i.e., switch performed after two repetitions) is associated with LH dominance. The lack of significant LH dominance in the no-change condition emphasizes the specialization of some LH areas in switching attention between levels. This result is consistent with the notion that LH areas have high level of proficiency governing the switching between levels (Fink et al., 1997a; Wilkinson et al., 2001; Schatz and Erlandson, 2003).

As expected, many repetitions of targets at the same level prior to a switch between levels increased the switch cost. In this study, variation in the number of previous level repetitions was aimed at specifically modulating the demands imposed to inhibiting the inappropriate level of analysis. When these demands increased, some aspects of the findings reflected the crucial role of RH areas. The RH dominance for this inhibitory process was reflected by the restriction of the detrimental effect of numerous level repetitions before switching within the RVF-LH. As illustrated in **Figures 5** and **6**, the lack of effect of the number of previous repetitions before switching in the LVF-RH cannot be interpreted as the function of a ceiling effect. Consequently, the RH appears to present a high level of proficiency in performing inhibition upon the inappropriate processing level. Additionally, a trend toward better accuracy in the LVF-RH than in the RVF-LH for level switches performed after four level repetitions was observed. By increasing the task difficulty, this high demanding condition provided opportunities to record behavioral evidence of hemispheric asymmetry in selective attention mechanisms (Evert and Oscar-Berman, 2001). In this condition, the attentional load was probably sufficiently demanding to require the best distribution of hemispheric involvement for the inhibition operation to be performed. Thus, the data converge on the notion of the crucial role of RH areas in inhibiting the inappropriate processing level. Since LH dominance was, in contrast, observed for the overall switching process, these clues for RH dominance when the switch strongly relied on inhibition revealed a reverse pattern of hemispheric asymmetry. This difference also confirmed the notion that the inhibitory mechanism can be specifically addressed among the switching process, as disengagement is separately assessed in spatial attention shifting (Posner, 1988).

The RH dominance when inhibiting the irrelevant processing level is consistent with the crucial role of right-lateralised areas in various forms of inhibition. The underlying neural networks may be different, but disengagement in spatial attention shifting is achieved in a most competent manner by a right cortical area (i.e., the right posterior parietal area; Robertson and Rafal, 2000). Additionally, task-switching experiments (changes between processing rules or judgment criteria are required to process a series of trials) also implicate one kind of internally mediated attentional switching and researchers have consistently emphasized the role of right-lateralised areas in inhibiting the inappropriate task-set when switching from one task to another one (Aron et al., 2004; Rogers et al., 2006). Similarly, response inhibition and the control of impulsivity is known to involve prefrontal and frontal-parietal networks preferentially in the RH (Aron et al., 2003; Rubia et al., 2003; Verbruggen et al., 2010). In the light of the consistent evidence for the major role of RH areas in various forms of inhibition, one potential interpretation of our pattern of results is that of evidence for the crucial role of RH areas in inhibiting information located at the inappropriate level or inhibiting the cognitive mechanisms involved in the inappropriate level of analysis of complex visual scenes*. *According to the *mechanism activation* hypothesis (Lamb and Yund, 1996b), each level of complexity is associated with specific neural mechanisms whose computations are not necessarily based on spatial frequency nor determined directly by the size of the attentional window, but are specific to the position of information within a hierarchical structure defined in terms of spatial hierarchical relations. Therefore, the level-repetition effect has been interpreted to occur at a relatively abstract stage of processing (Hübner, 2000). This may also be the case for the switch cost and the specific inhibitory mechanism assessed in our experiment.

The study has a few limitations that should be considered. One, although all the stimulus category and visual field effects pointed in the same expected directions when recorded on RTs and on ERs, the effects sometimes reached significance only for one of the outcome variables. Two, functional hemispheric asymmetries were investigated by using tachistoscopic lateralized presentation of visual stimuli, which has been shown to reliably reveal functional differences between the two hemispheres. However, a more precise localisation of the cerebral areas involved in the inhibitory process assessed in this study should be considered in future investigations, either by using brain imagery techniques or by observing the patterns of performance of patients with specific cerebral lesions. Nevertheless, the present data provide both new evidence regarding the role of both the hierarchical level of information and the stimulus category in elicitingthe involvement of right and LH areas in processing complex visual scenes and in switching between global and local levels of complex visual stimuli.

## AUTHOR CONTRIBUTIONS

Luc Kéïta and Nathalie Bedoin designed experiments. Luc Kéïta, Nathalie Bedoin, Jacob A. Burack, and Franco Lepore wrote the main manuscript text. Luc Kéïta, Nathalie Bedoin, and Franco Lepore analyzed the data. Luc Kéïta and Nathalie Bedoin prepared figures. All authors (Luc Kéïta, Nathalie Bedoin, Jacob A. Burack, and Franco Lepore) reviewed the manuscript. Franco Lepore paid the manuscript fee.

## Conflict of Interest Statement

The authors declare that the research was conducted in the absence of any commercial or financial relationships that could be construed as a potential conflict of interest.
